# An adverse outcome pathway-aligned ex vivo mouse testicular organ culture platform for mechanistic integration of multi-level endpoints with recovery assessment

**DOI:** 10.1093/toxsci/kfag037

**Published:** 2026-03-28

**Authors:** Hideaki Nakagiri, Kyoichi Kodama, Shimpei Terasaka, Makoto Kashima, Naohiro Ikeda, Yuko Nukada, Kenkichi Fujii, Yoshikazu Nagao

**Affiliations:** Department of Animal Production Science, United Graduate School of Agricultural Science, Tokyo University of Agriculture and Technology, Fuchu, Tokyo, Japan; University Farm, Faculty of Agriculture, Utsunomiya University, Tochigi, Japan; Strategy Promotion and Administration Center, Regulatory Science, Kao Corporation, Tokyo, Japan; Safety Science Research Laboratories, Kao Corporation, Haga, Tochigi, Japan; Safety Science Research Laboratories, Kao Corporation, Haga, Tochigi, Japan; Department of Biomolecular Science, Faculty of Science, Toho University, Funabashi, Japan; Safety Intelligence & Research, Product Quality Management, Kao Corporation, Tokyo, Japan; Safety Science Research Laboratories, Kao Corporation, Haga, Tochigi, Japan; Strategy Promotion and Administration Center, Regulatory Science, Kao Corporation, Tokyo, Japan; Department of Animal Production Science, United Graduate School of Agricultural Science, Tokyo University of Agriculture and Technology, Fuchu, Tokyo, Japan; University Farm, Faculty of Agriculture, Utsunomiya University, Tochigi, Japan

**Keywords:** male reproductive toxicity, adverse outcome pathway (AOP), spermatogenesis, testicular organ culture, PDMS ceiling method

## Abstract

Mechanistic understanding is important for improving the safety assessment of male reproductive toxicity; however, current evaluations still rely primarily on in vivo studies. Therefore, the development of new approach methodologies (NAMs) requires test systems capable of capturing key events organized within adverse outcome pathways (AOPs). The PDMS ceiling (PC) method is a testicular organ culture technique previously used to detect testicular toxicity. However, its applicability as an AOP-aligned platform for multi-level mechanistic assessment, including recovery evaluation, has not been fully examined. Here, we evaluated the utility of the PC-based organ culture system for AOP-aligned assessment of testicular toxicity. Methoxyacetic acid (MAA), a testicular toxicant with established AOPs, was used as a reference compound. Molecular, cellular, and histological endpoints were assessed under identical conditions, and a membrane-supported PC (msPC) configuration was introduced to enable controlled recovery assessment. Testes from Acro3-EGFP transgenic mice, in which EGFP expression driven by the acrosin promoter accumulates in developing acrosomes, were cultured and exposed to MAA. GFP fluorescence indicating spermatogenic progression decreased in a concentration-dependent manner. Histological and immunohistochemical analyses demonstrated selective loss of pachytene spermatocytes with apoptosis, whereas transcriptomic profiling suggested disruption of cell cycle- and meiosis-related pathways consistent with established AOP-defined key events. Recovery of GFP fluorescence and tissue morphology occurred earlier in the msPC system than in the conventional PC method. These findings support the PC-based organ culture system as an AOP-aligned platform within NAMs for evaluating testicular toxicity and recovery.

Infertility is a major global public health concern, affecting approximately one in six individuals worldwide (World Health Organization 2023), with male factors contributing to 30% to 50% of cases either independently or in combination with female factors, largely through impairment of spermatogenesis, a principal underlying mechanism of male infertility (Cox et al. 2022; Eisenberg et al. 2023). Because chemical perturbations disrupt spermatogenesis in a manner dependent on germ cell differentiation and supporting somatic cell function (Skakkebaek et al. 2016), interpretation of testicular toxicity requires reproducible, mechanism-based experimental systems.

In parallel with these scientific needs, new approach methodologies (NAMs) have been proposed to reduce or complement testing involving intact animals (U.S. Environmental Protection Agency 2021; Organisation for Economic Co-operation and Development (OECD) 2023). When applied to reproductive and testicular toxicity, NAM-based approaches are expected to support mechanism-based toxicity assessment while addressing ethical and practical limitations of in vivo testing (Tanabe et al. 2021; Organisation for Economic Co-operation and Development (OECD) 2020). However, despite the availability of ex vivo systems capable of reproducing testis-specific processes such as meiosis and spermatogenesis (Sato et al. 2011; Komeya et al. 2017), experimental implementation of NAMs for testicular toxicity remains limited, particularly with respect to integrating mechanistic information across biological levels within a single experimental system.

A central challenge in advancing NAMs for testicular toxicity lies in the need for a conceptual framework that can organize mechanistic information in a biologically and temporally coherent manner. The adverse outcome pathway (AOP) framework addresses this need by organizing toxicity mechanisms from molecular initiating events (MIEs) through key events (KEs) to adverse outcomes (AOs), supporting mechanistic extrapolation and benchmark dose derivation (Organisation for Economic Co-operation and Development (OECD) 2018). However, whether such mechanistic progression can be traced experimentally over time within the same tissue–particularly dynamically differentiating and recovering tissues such as the testis—remains a practical challenge.

In the testis, toxic effects often emerge as temporally ordered disruptions of spermatogenesis (Chapin et al. 1985; Zhang et al. 2024). Because such responses are time-dependent and dependent on germ cell differentiation and supporting somatic cell function, experimental establishment of causal linkages among AOP-defined key events is inherently challenging. Therefore, it remains unclear whether existing organ culture systems can sequentially link multiple AOP-defined KEs while enabling assessment of post-exposure recovery processes in a manner suitable for regulatory NAM applications within a single experimental framework.

In our study, we utilized Acro3-EGFP transgenic mice, in which, enhanced green fluorescent protein (EGFP) expression driven by the proacrosin promoter becomes detectable from the mid-pachytene stage onward, enabling time-resolved visualization of meiotic progression and spermiogenesis (Nakanishi et al. 1999; Ventelä et al. 2000). These features suggest that molecular-, cellular-, and tissue-level perturbations could, in principle, be assessed within a single experimental framework; however, whether AOP-defined mechanistic progression, including recovery, can be operationalized in practice remains insufficiently examined.

Among available NAM-compatible culture techniques, the PDMS-ceiling (PC) method employs an oxygen-permeable polydimethylsiloxane (PDMS) chip to suppress tissue aggregation and ensure uniform oxygen and nutrient supply to flattened tissue fragments, improving structural uniformity, reproducibility, and optical accessibility for live imaging (Komeya et al. 2017). This configuration enables time- and dose-dependent acquisition of molecular-, cellular-, and tissue-level endpoints following toxicant exposure within a single system (Nakagiri et al. 2024), thereby providing a practical basis for aligning upstream molecular changes with downstream cellular- and tissue-level KEs in the context of the AOP framework.

Nevertheless, most toxicity studies using the PC method have focused on histological outcomes (Hashimoto et al. 2024; Yokota et al. 2025), and sequential analyses of KEs and key event relationships (KERs) across biological levels remain limited. Furthermore, although assessment of toxicity reversibility is critical for mechanistic interpretation and regulatory decision-making (International Council for Harmonisation of Technical Requirements for Pharmaceuticals for Human Use (ICH) 2011; Hukkanen et al. 2023), strict control of postexposure conditions is not always feasible in organ culture systems. In agarose-supported organ culture configurations, including the PC method, chemicals may persist within the gel matrix in contact with the tissue, leading to overexposure that can confound evaluation of recovery processes and interpretation of dose-response relationships.

Therefore, to establish PC-based testicular organ culture as a reliable NAM platform for mechanism-based toxicity assessment, it is necessary to determine whether this system can capture sequential KEs, coherent KERs, and cell-type-specific toxic responses, while also enabling evaluation of recovery under conditions in which effective exposure after treatment cessation is appropriately controlled. Methoxyacetic acid (MAA), a well-characterized histone deacetylase inhibitor known to induce pachytene spermatocyte apoptosis followed by testicular atrophy (Tanabe et al. 2021), serves as an appropriate probe compound for examining AOP-aligned KE progression and recovery dynamics. Here, we apply the AOP concept to establish and evaluate a mouse testis organ culture system using the PC method to characterize time- and dose-dependent toxicity across biological levels, and to analyze transitions from toxicity onset to recovery.

## Materials and methods

### Preparation of PC chips

**Fig. 1.**
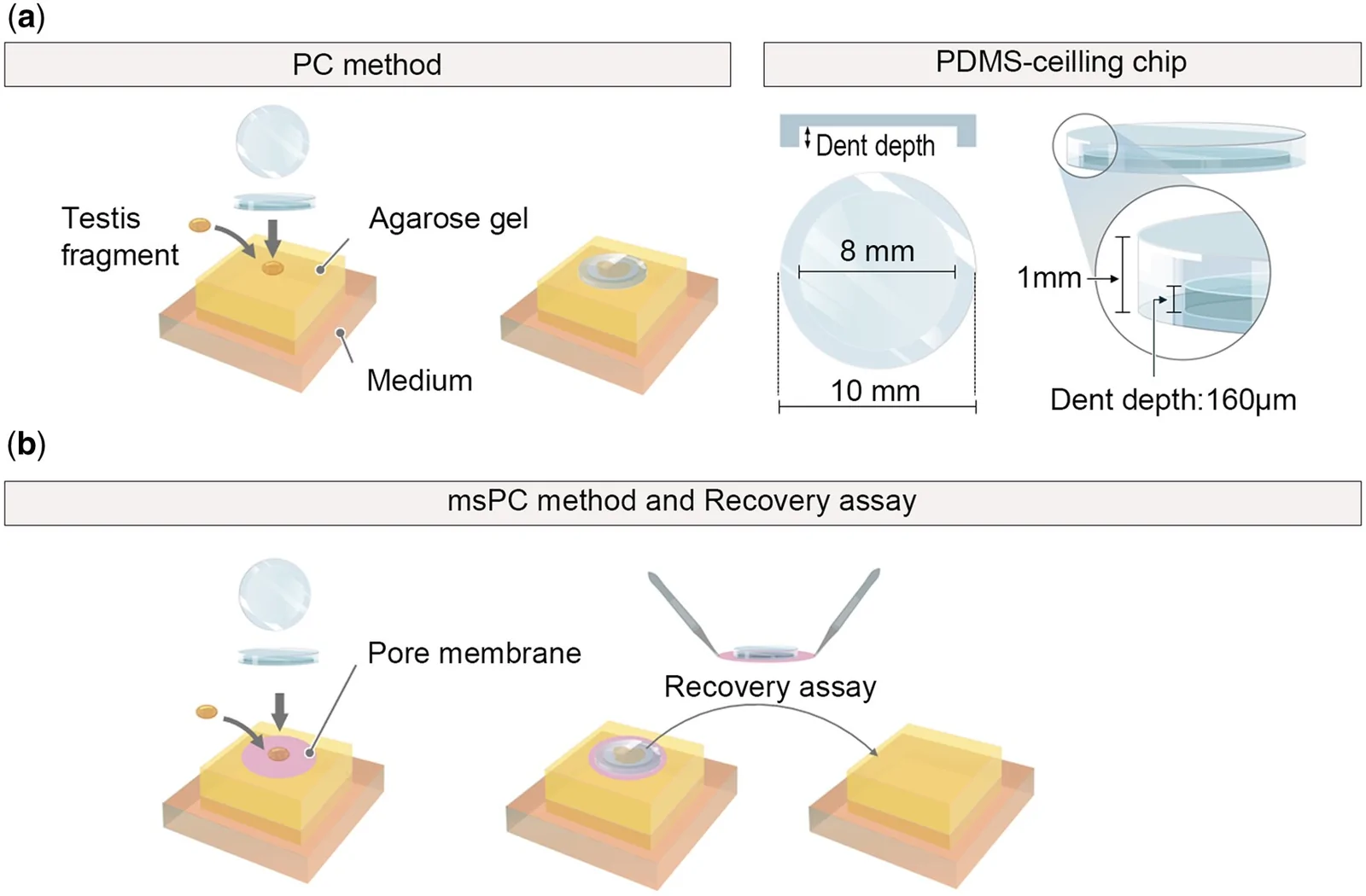
Schematic illustration of the PC and msPC culture methods and the experimental workflow for chemical exposure and recovery assays. (a) PDMS-ceiling (PC) method, in which testis tissue fragments are cultured in a flattened configuration under a recessed PDMS chip placed on half-immersed agarose gel blocks. (b) Membrane-supported PDMS-ceiling (msPC) method, in which tissue fragments are positioned on a polycarbonate membrane and covered with a PDMS chip, allowing easy transfer during recovery assays.

The fabrication and specifications of the PC chips were based on previous studies (Komeya et al. 2019; Nakagiri et al. 2024). Chips (Fukoku Bussan Co., Ltd., Tokyo, Japan) were prepared from liquid silicone rubber using transfer molding and photolithography techniques and featured a diameter of 10 mm with a circular dent of 8 mm in diameter and 160 µm in depth (Fig. 1a). All the chips were autoclaved before use.

### Animals

Testis tissues were obtained from a transgenic mouse line expressing enhanced green fluorescent protein (EGFP) under the control of the proacrosin promoter (BRC No.: RBRC00886; strain name: B6; B6C3-Tg(Acro3-EGFP)01Osb) provided by RIKEN BRC through the National BioResource Project (MEXT/AMED, Japan) (Nakanishi et al. 1999; Ohta et al. 2009). In this manuscript, this registered transgenic line is hereafter referred to as Acro3-EGFP mice. In this model, EGFP expression driven by the proacrosin promoter becomes detectable from the mid-pachytene stage onward, enabling visualization of meiotic progression and spermiogenesis. Transgene-positive offspring were bred on a C57BL/6 background, and homozygous males at 5 to 7 d postpartum (dpp) were used for culture experiments. A total of 16 male mice (5 to 7 dpp) were used in this study. Mice were housed at the Kao Corporation animal facility under controlled conditions (22 ± 1°C, 40% to 60% humidity, 12 h light/dark cycle) with food and water available ad libitum. All experimental procedures were performed under isoflurane anesthesia, and mice were euthanized by cervical dislocation under anesthesia at the end of the experiments. Humane endpoints were predefined, and animals were monitored at appropriate intervals. All procedures were approved by the Animal Care Committee of Kao Corporation (approval no. F23010-0001, S22069-0002, and S23011-0001) and conducted in accordance with institutional guidelines, the Act on Welfare and Management of Animals (Japan), and the ARRIVE guidelines.

### Testis organ culture

As previously reported (Kojima et al. 2018; Komeya et al. 2019), testis tissue fragments from 5 to 7 dpp mice were cultured on half-immersed agarose gel blocks (1.5% w/v, ∼1.25 cm^2^) in α-Minimum Essential Medium (α-MEM) supplemented with AlbuMAX (40 mg/ml), sodium bicarbonate (2.6%), and antibiotic-antimycotic. Decapsulated testes were divided into approximately 6 to 8 fragments and placed in 12-well plates for PDMS-ceiling (PC) and membrane-supported PC (msPC) cultures. Cultures were maintained at 34°C in an incubator supplied with 5% CO_2_.

#### PC method

For the PC method, a PDMS ceiling chip was placed on each tissue fragment with the recessed side facing downward while the fragment rested on an agarose block (Fig. 1a).

#### msPC method

We developed an msPC system in which testis fragments were positioned between a polycarbonate membrane and a PDMS ceiling chip. A 0.4-µm membrane sheet (Isopore, Merck Millipore; HTTP01300) was placed on a half-immersed agarose block. The chip was set above the tissue with its recessed surface facing downward (Fig. 1b). This configuration enabled easy handling of the membrane-tissue-chip assembly for transfer and recovery assays.

### Experimental design

#### MAA exposure

Testis tissue fragments from 5- to 7-d-old Acro3-EGFP mice were cultured for 4 wk to allow stable GFP expression and spermatogenic differentiation before MAA exposure (Nakagiri et al. 2024). Tissue fragments were exposed to methoxyacetic acid (MAA; 1 to 4 mM) for 24 h, with distilled water used as the vehicle control. The concentration range was selected based on previous in vivo-in vitro studies reporting seminiferous tubule toxicity at millimolar MAA concentrations (Ku and Chapin 1994; Antonine et al. 2022). GFP fluorescence and bright-field images were acquired before and after exposure. Exposure experiments were performed using nine to ten independent samples per treatment group. Of these, six samples were subjected to immunohistochemical analysis and three to four to PAS staining. Tissues were fixed for periodic acid-Schiff (PAS) staining or immunohistochemistry (IHC).

#### Recovery assay

The experimental setup and workflow for the recovery assay are shown in Figs 1b and 7a. Testis tissue fragments cultured for 4 wk using the msPC method were exposed to 4 mM MAA for 24 h. After exposure, the membrane-tissue-chip assembly was transferred to agarose gel blocks pre-equilibrated with fresh medium. In the PC method, MAA-containing medium was removed and agarose gel blocks were washed three times at 0, 2, 4, and 6 h and at 1, 2, and 4 d post-exposure to eliminate residual MAA. Tissues cultured using both methods were monitored during the recovery period, collected on day 9, and subjected to histological evaluation. Recovery experiments were performed using eight independent samples per treatment group; of these, five were subjected to immunohistochemical analysis and three to PAS staining. Residual MAA levels in agarose gels were quantified separately.

### Observation of cultured tissues

GFP expression and bright-field images of the cultured tissues were acquired periodically using a stereomicroscope (Leica M205 FA; Leica). During microscopic observation, the culture plate was sandwiched between the upper and lower plates of a ThermoPlate (ThermoPlate III; Cell Seed Inc., CST008) to maintain an appropriate temperature and prevent condensation on the lid from interfering with image acquisition.

### RNA-seq library preparation and bioinformatic processing

Total RNA was extracted from cultured testis tissues (n = 4 to 5 per group) using the RNeasy Mini Kit (74104, Qiagen, Hilden, Germany) according to the manufacturer’s instructions. RNA quality was assessed by spectrophotometry, and samples with an A260/280 ratio between 1.8 and 2.0 were used for library preparation.

RNA-seq libraries were prepared using the low-cost and easy RNA-seq (Lasy-Seq) method (version 1.1) as previously described (Kamitani et al. 2019; Lasy-Seq protocol site 2019), which employs oligo(dT)-primed reverse transcription to selectively capture polyadenylated RNA molecules. DNase treatment was not performed.

Briefly, first-strand cDNA synthesis and library construction were performed following the Lasy-Seq v1.1 protocol (https://sites.google.com/view/lasy-seq/). For library construction, 135 ng of total RNA per sample was used as input. Libraries were sequenced using 150-bp paired-end reads on a NovaSeq X plus platform (Illumina, San Diego, CA, USA).

Raw sequencing reads were processed using fastp (version 0.21.0) to remove low-quality bases and adapter sequences. The following parameters were applied: Trimming of poly-X tails (-trim_poly_x), window-based quality trimming (-w 20), and adapter removal using the sequences AGATCGGAAGAGCACACGTCTGAACTCCAGTCA and AGATCGGAAGAGCGTCGTGTAGGGAAAGAGTGT, with a minimum read length of 31 bp. Processed reads were aligned to the Mus musculus reference genome (GRCm38) using bwa mem (version 0.7.17-r1188). Transcript quantification was performed using salmon (version 0.12.0), specifying the library type as −l IU.

Downstream statistical analyses were conducted in R (version 4.5.1). Differential expression analysis was performed using the TCC package (version 1.48.0) (Sun et al. 2013). Genes showing statistically significant changes in expression were identified based on false discovery rate (FDR)-adjusted *P*-values, with an FDR threshold of 0.05. Gene Ontology (GO) enrichment analysis was conducted using the clusterProfiler package (version 4.16.0) (Wu et al. 2021).

### Histological and immunohistochemical examinations

Cultured testis tissues were fixed in Bouin’s fixative, embedded in paraffin, and sectioned horizontally at a thickness of 6 µm to obtain the largest cross-sectional area. Sections were subjected to periodic acid-Schiff (PAS) staining for histological evaluation.

For immunohistochemistry (IHC), cultured tissues were fixed in 10% neutral buffered formalin (133-10311, FUJIFILM Wako Pure Chemical Corporation) at 4°C overnight, embedded in paraffin, and sectioned horizontally at a thickness of 6 µm. Deparaffinized and rehydrated sections were treated with antigen retrieval buffer (pH 9.0; S2367, DAKO) at 98°C for 10 min and allowed to cool to room temperature.

To block nonspecific binding, sections were incubated with normal goat serum (426042; Nichirei Biosciences Inc.) for 1 h at room temperature. Sections were then incubated with primary antibodies overnight at 4°C. After washing with PBS, sections were incubated for 1 h at room temperature in the dark with secondary antibodies diluted in PBS containing 4′,6-diamidino-2-phenylindole (DAPI; 1:400, D523; Dojindo, Kumamoto, Japan). Sections were washed several times with PBS, mounted using mounting medium (0100-01, Southern Biotechnology Associates, Inc.), and placed on raised coverslips.

The primary antibodies used for IHC were anti-SALL4 (1:250, ab57577, Abcam), anti-α-smooth muscle actin (α-SMA)-Alexa Fluor 647 (1:200, ab202296, Abcam), anti-Wilms’ tumor 1 (WT1; 1:250, MAB4234, Millipore), anti-synaptonemal complex protein 3 (SYCP3; 1:400, ab97672, Abcam), and anti-γH2AX (1:400, 9718, Cell Signaling Technology). SALL4, WT1, and α-SMA were used as markers for spermatogonia, Sertoli cells, and peritubular myoid cells, respectively (Chan et al. 2017; Rotgers et al. 2014; Kawabe et al. 2022). PAS staining was evaluated in three to four independent samples per treatment group. Immunohistochemical analyses were performed in six independent samples per treatment group.

### TUNEL assay

Apoptotic cells were detected using the In Situ Cell Death Detection Kit, TMR Red (12156792910; Merck), following the manufacturer’s instructions. After TUNEL labeling, sections were incubated with Alexa Fluor 647-conjugated anti-α-smooth muscle actin (α-SMA) antibody (1:200, ab202296, Abcam) and DAPI (1:400, D523; Dojindo, Kumamoto, Japan) for 1 h at room temperature in the dark. Sections were washed with PBS, mounted using a commercial mounting medium (0100-01, Southern Biotechnology Associates, Inc.), and imaged with an inverted fluorescence microscope (BZ-X700; KEYENCE, Osaka, Japan). Four independent samples were analyzed per treatment group.

### MAA analysis in agar by LC-MS/MS

The concentration of methoxyacetic acid (MAA) within agarose gel blocks was quantified using a modified method based on Ning et al. (2018). Agarose gel blocks containing 4 mM MAA were washed according to the recovery assay protocol using identical time points and numbers of washes. Measurements were performed using three independent samples at each time point. At each time point, gel blocks were processed using a QIAshredder spin column (cat. no. 79656). Gel pieces were placed on the column, centrifuged at 20,000 × g for 15 min, and the eluates were collected.

The eluates were diluted 200-fold with water. Aliquots (100 µl) of the diluted samples were mixed with 900 µl of acetonitrile and centrifuged at  20,000× g for 10 min at 4°C. A total of 500 µl of the supernatant was collected and evaporated to dryness under nitrogen. The residues were reconstituted in 100 µl of water containing ranitidine (0.001 µM; TCI, R0261) as an internal standard (IS).

MAA was quantified using an Exion LC-AD system coupled with a QTRAP 5500 mass spectrometer (SCIEX, Framingham, MA, USA). Chromatographic separation was achieved using a Poroshell 120 EC-C18 column (2.7 µm, 4.6 × 50 mm; Agilent Technologies) maintained at 40°C. The mobile phase consisted of 10 mM ammonium formate (A) and acetonitrile containing 0.1% formic acid (B). A gradient elution at a flow rate of 0.5 ml/min was applied as follows: 0.00 min, 0% B; 0.50 min, 0% B; 1.50 min, 7% B; 2.00 min, 45% B; 3.60 min, 45% B; 3.61 min, 95% B; 4.20 min, 95% B; 4.21 min, 0% B; 5.00 min, 0% B.

The mass spectrometer was operated using electrospray ionization (ESI). MAA was analyzed in negative-ion mode and ranitidine in positive-ion mode using multiple reaction monitoring (MRM), with transitions of m/z 89.0 → 74.2 for MAA and m/z 315.4 → 176.0 for ranitidine. The ion spray voltage was set to +4,100 V and −4,100 V, and the source temperature was 600°C. Additional parameters were as follows: Declustering potential, −40 V for MAA and 41 V for ranitidine; entrance potential, −10 V and 10 V; collision energy, −12 V and 25 V; and collision cell exit potential, −10.5 V and 14 V, respectively. Sample injections (2 µl) were performed using an autosampler maintained at 4°C.

### Quantitative image analysis

Bright-field and GFP fluorescence images of cultured tissues were acquired using a fluorescence stereomicroscope (Leica M205 FA; Leica) and analyzed with the Hybrid Cell Count application (BZ-H4C, Keyence) integrated into BZ-X Analyzer software (BZ-H4A, Keyence). The horizontal projection area of each cultured tissue was quantified from bright-field images, and the GFP-positive area within the same tissue was quantified and expressed as a ratio to the corresponding horizontal projection area.

To quantify the total number of spermatocytes and those at each meiotic phase, 4 to 6 nonoverlapping fields were systematically acquired from each tissue section. Fields were selected in a random and unbiased manner to avoid intentional selection bias. Within these fields, at least 45 seminiferous tubules per tissue were examined.

TUNEL-positive areas and immunohistochemically positive areas (including SALL4-positive nuclei) were quantified in four nonoverlapping fields per section (Nakagiri et al. 2024). WT-1-positive Sertoli cells were counted manually in four nonoverlapping fields per section. For TUNEL analysis, the positive area was calculated relative to the tissue section area of the cultured tissue fragment within each analyzed field of view. Values were expressed as relative percentages normalized to the vehicle control (set to 100%).

For all figures, representative images were selected from among the quantified images as those whose quantitative values were closest to the group mean.

### Statistical analysis

Data are presented as mean ± SD unless otherwise specified. For group-wise comparisons, data are presented as box-and-whisker plots or individual data points where appropriate. For longitudinal time-course analyses, results are presented as mean ± SD to illustrate temporal trends. Statistical analyses were performed using GraphPad Prism (version 8.0; GraphPad Software, San Diego, CA, USA). One-way ANOVA was used for group comparisons, followed by the Tukey-Kramer test for post hoc analyses. A value of *P *< 0.05 was considered statistically significant.

## Results

### Early GFP-defined spermatogenic impairment precedes tissue atrophy after exposure

**Fig. 2.**
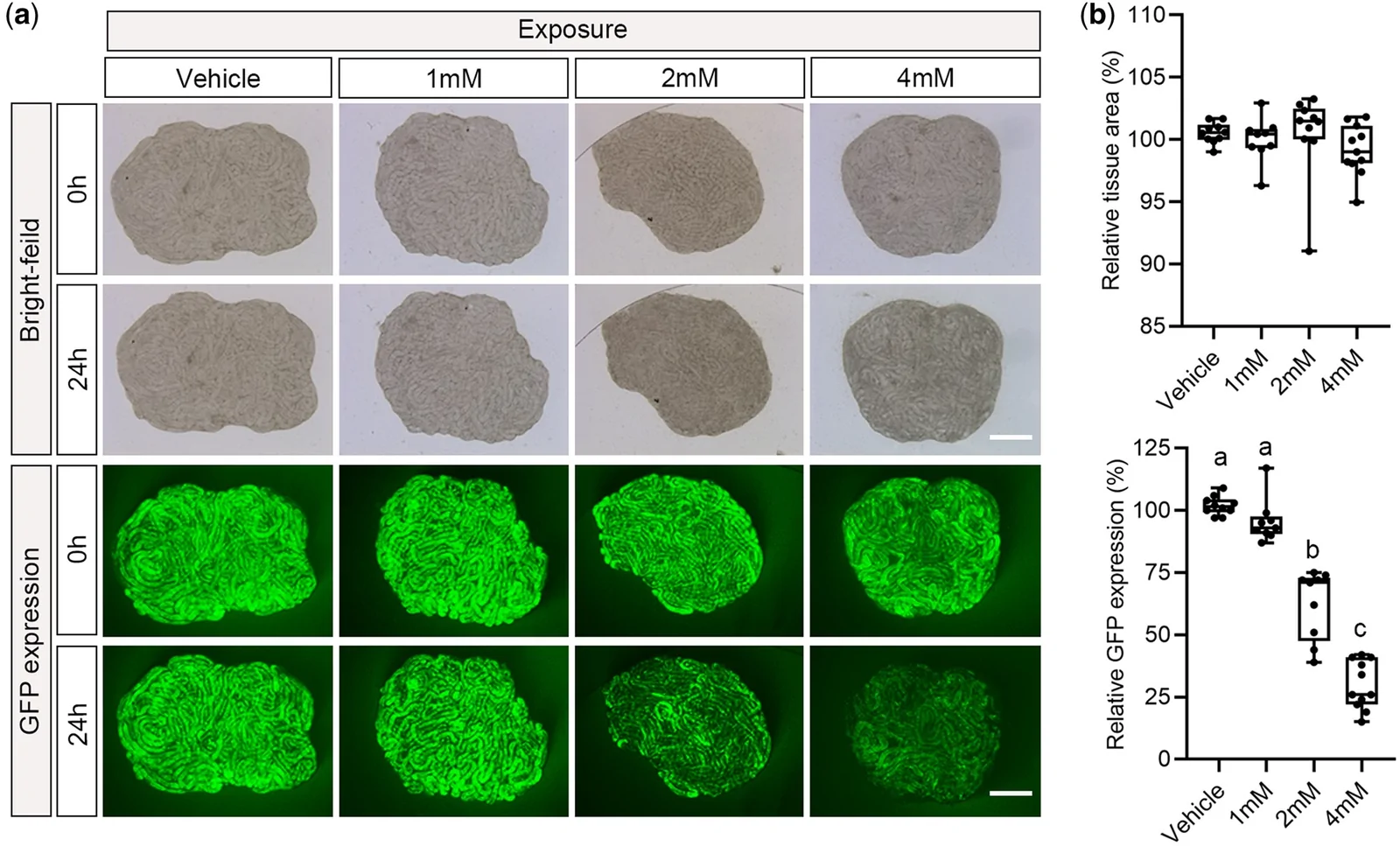
Stereomicroscopic and fluorescence readouts of cultured testes after exposure. Cultured testis tissues were exposed to methoxyacetic acid (MAA), used as a mechanistically well-characterized reference compound, for 24 h, and bright-field and GFP fluorescence images were obtained together with quantitative analysis of tissue area and GFP-positive area. (a) Representative bright-field and GFP fluorescence images after 24 h exposure to vehicle or 1 to 4 mM MAA. Scale bar: 1 mm. (b) Quantification of horizontally projected tissue area and GFP-positive area expressed as relative values normalized to their respective pre-exposure levels (0 h = 100%). Projected tissue area did not differ among groups, whereas GFP-positive area decreased in a concentration-dependent manner. Data are shown as box-and-whisker plots (*n* = 9 to 10). One-way ANOVA with Tukey’s test; different letters indicate *P* < 0.05.

To identify key events that occur prior to the manifestation of overt tissue atrophy as an adverse outcome, we analyzed early spermatogenic impairment in testicular tissues cultured using the PC method under identical experimental conditions. Live stereomicroscopic imaging was performed before exposure and after 24 h of MAA treatment (Fig. 2a).

In Acro3-EGFP transgenic mice, GFP expression becomes detectable from mid-pachytene spermatocytes onward and persists thereafter, enabling monitoring of changes in cell populations that reflect late meiotic progression. For quantitative analysis of stereomicroscopic images, both the horizontally projected tissue area calculated from bright-field images and the GFP-positive area were normalized to each tissue’s pre-exposure baseline (0 h set to 100%) (Fig. 2b).

At this time point, no significant change in projected tissue area was observed in any treatment group. In contrast, while the GFP-positive area remained stable in vehicle-treated controls, it was significantly reduced in the 2- and 4-mM MAA-exposed groups in a concentration-dependent manner (Fig. 2b). These findings demonstrate a concentration-dependent reduction in GFP-positive area under the present experimental conditions.

### Histological features of spermatocyte loss underlying GFP-defined early responses

**Fig. 3.**
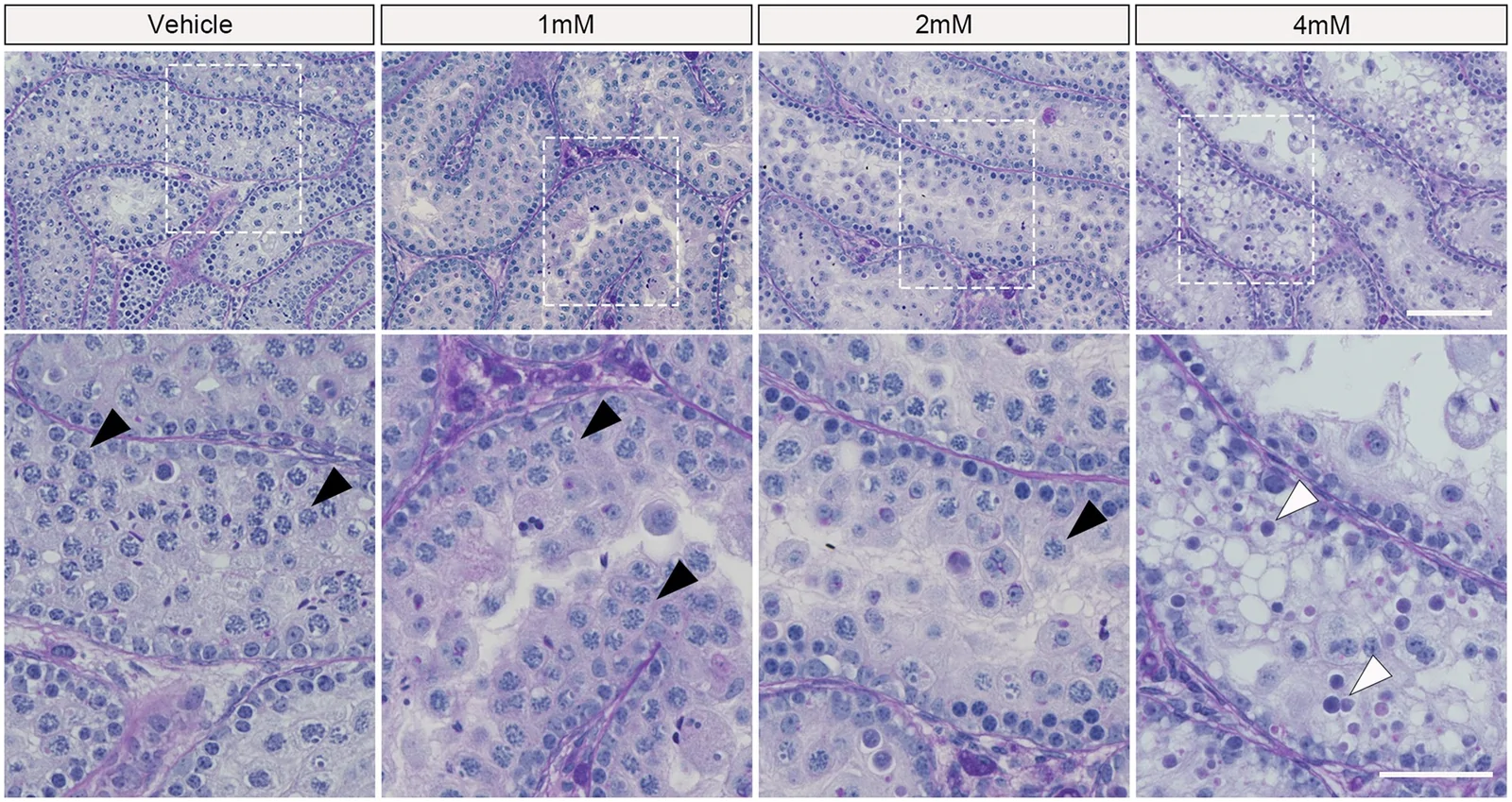
Histological features of cultured testis tissues after exposure. PAS-stained sections of cultured testis tissues obtained 24 h after exposure to vehicle or 1 to 4 mM MAA. Regions enclosed by dashed boxes in the upper panels are shown at higher magnification in the lower panels. Partial loss of spermatocytes (black arrowheads) was observed at 2 mM, with more extensive depletion at 4 mM accompanied by degenerative-like morphological changes (white arrowheads). Vehicle-treated cultures largely maintained overall seminiferous tubule architecture, although occasional apoptotic cells consistent with baseline culture-associated changes were observed. Scale bars: 40 µm (upper panels), 80 µm (lower panels). Representative images from three to four independent samples per treatment group are shown.

Representative sections obtained after 24 h of MAA exposure are shown in Fig. 3. In the preceding analysis, a concentration-dependent reduction in the GFP-positive area was identified as an early response to MAA exposure. To understand the histological basis of this GFP reduction, histological examination was performed to evaluate spermatocyte loss in the cultured testicular tissues. PAS staining was used to visualize seminiferous tubule architecture and germ cell morphology. Under identical exposure conditions, the distribution of spermatocytes in the 1-mM MAA group was comparable to that observed in vehicle-treated controls. In contrast, the 2-mM group exhibited a noticeable reduction in spermatocytes, whereas the 4-mM group showed a pronounced loss of spermatocytes accompanied by apoptotic-like morphological changes. Minor structural irregularities and occasional apoptotic cells were observed in vehicle-treated cultures, which are inherent to ex vivo organ culture conditions. However, these features were qualitatively and quantitatively distinct from the concentration-dependent alterations induced by MAA exposure.

These histological alterations occurred in a concentration-dependent manner at the same time point at which GFP-defined impairment was observed.

### Phase-specific depletion of spermatocytes reveals selective vulnerability after exposure

**Fig. 4.**
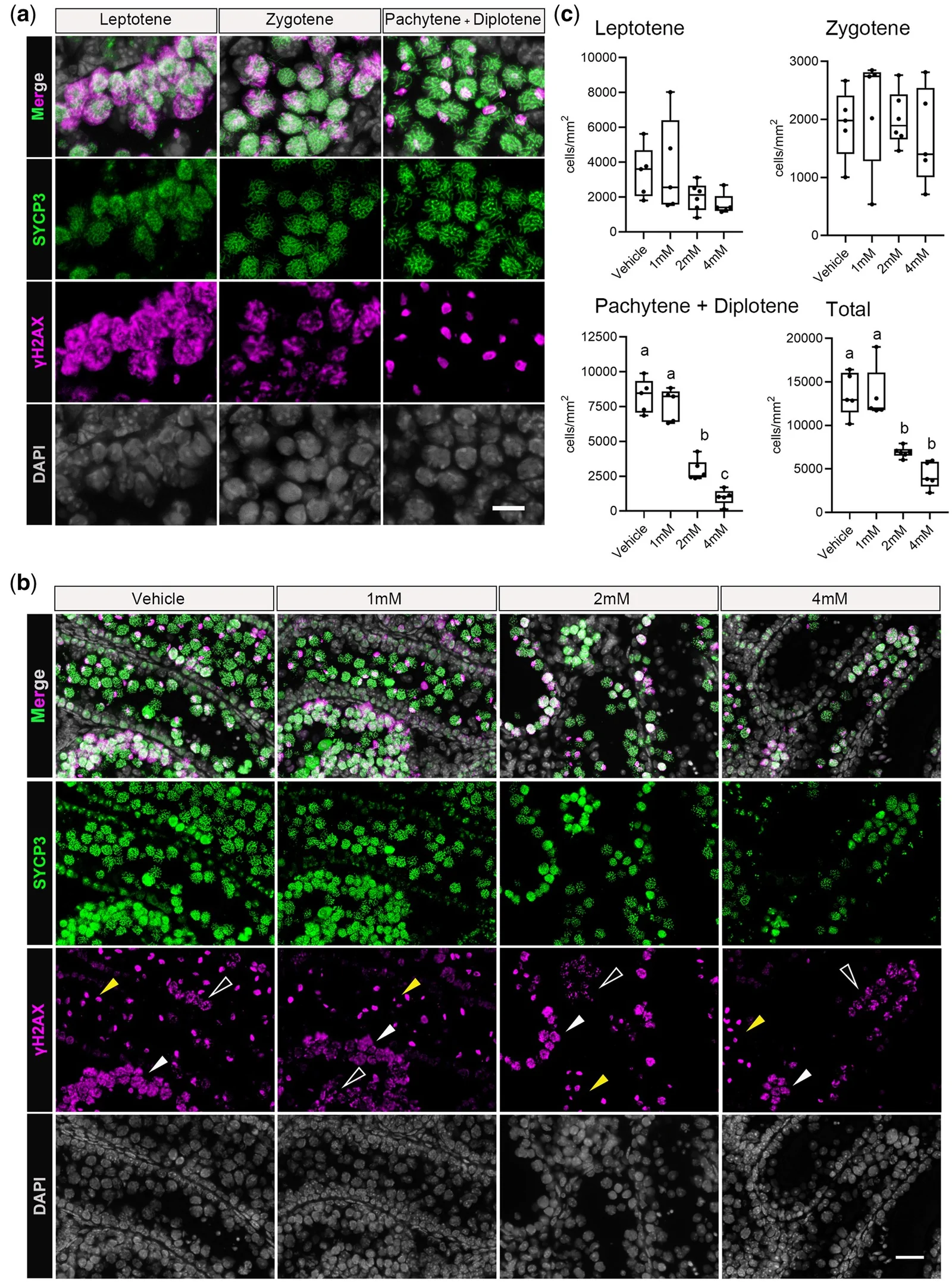
Phase-resolved analysis of spermatocyte populations in testis organ culture. (a) Representative immunofluorescence images defining meiotic phases of spermatocytes based on γH2AX and SYCP3 staining. Spermatocytes were classified into leptotene, zygotene, and pachytene/diplotene stages according to established marker patterns and nuclear morphology (Fig. S1). (b, c) Quantification of spermatocyte numbers by meiotic phase after 24 h exposure. Data are shown as box-and-whisker plots (n = 6). One-way ANOVA with Tukey’s test; different letters indicate *P* < 0.05. SYCP3, green; γH2AX, magenta; DAPI, gray. Scale bars: 20 µm (a), 40 µm (b).

An overview of meiotic phase classification and immunohistochemical analyses is shown in Fig. 4a-c. In the preceding histological analysis, spermatocyte depletion was identified as an early cellular- and tissue-level response to exposure; however, the meiotic phases underlying this response within the same experimental framework remained unclear. To clarify how spermatocyte depletion—manifested as selective loss of pachytene spermatocytes—emerges under identical conditions, we examined phase-specific sensitivity using immunohistochemistry in the PC method-based testis organ culture system. γH2AX and SYCP3 immunostaining was used to classify spermatocytes into leptotene, zygotene, and combined pachytene/diplotene stages (Ribagorda et al. 2019; Wakayama et al. 2022). This classification scheme was validated in vivo (Fig. S1) and then applied in the same manner to organ-cultured testicular tissues (Fig. 4a).

Representative immunohistochemical images after exposure are shown in Fig. 4b, and quantitative analyses are presented in Fig. 4c. Under identical exposure conditions and at the same time point used to define early spermatogenic impairment, the number of pachytene spermatocytes was significantly reduced in the 2- and 4-mM groups in a concentration-dependent manner, accompanied by a decrease in the total number of spermatocytes. In contrast, no significant changes were detected in spermatogonia or Sertoli cells (Fig. S2).

### Apoptotic germ-cell responses accompanying early spermatocyte depletion after exposure

Histological and phase-specific immunohistochemical analyses demonstrated selective depletion of spermatocytes following MAA exposure. To examine whether this depletion was accompanied by apoptotic germ-cell death within the same organ culture system, apoptosis was assessed using TUNEL staining as a cellular-level response.

**Fig. 5.**
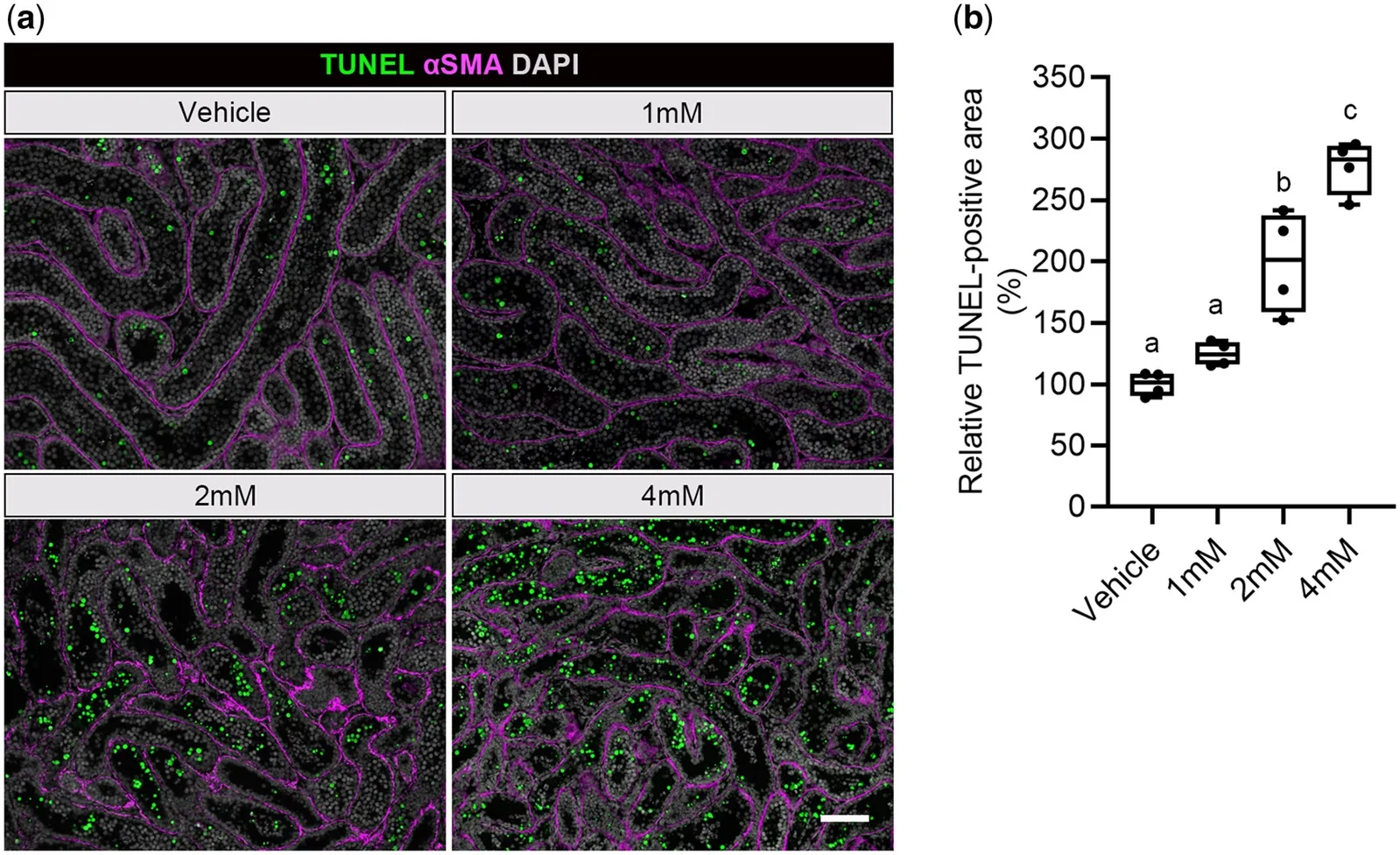
Detection of apoptotic responses in testis organ culture after exposure. (a) Representative TUNEL-stained images obtained 24 h after exposure to vehicle or 1 to 4 mM MAA. Scale bars: 80 µm. (b) Quantification of TUNEL-positive areas expressed relative to vehicle controls. Data are shown as box-and-whisker plots (*n* = 4). One-way ANOVA with Tukey’s test; different letters indicate *P *< 0.05. TUNEL, green; DAPI, gray; αSMA, magenta. αSMA staining was used to delineate seminiferous tubule boundaries.

Representative TUNEL-stained images and quantitative analyses of organ-cultured testicular tissues exposed to increasing concentrations of MAA for 24 h are shown in Fig. 5a and b. Because TUNEL-positive nuclei frequently appeared fragmented, the TUNEL-positive area was used for quantification and expressed as a relative value, with the vehicle control set to 100%.

Under identical exposure conditions, the relative TUNEL-positive area increased in a concentration-dependent manner, with significant increases observed at concentrations of 2 mM or higher. TUNEL-positive signals were predominantly localized to the upper layers of the seminiferous epithelium, consistent with the spatial distribution of spermatocytes.

At this time point, apoptotic germ-cell responses were observed in the same testis organ culture system in which GFP-defined spermatogenic impairment and histological spermatocyte depletion were detected.

### Transcriptomic profiling reveals early molecular perturbations and a candidate intermediate event after exposure

**Fig. 6.**
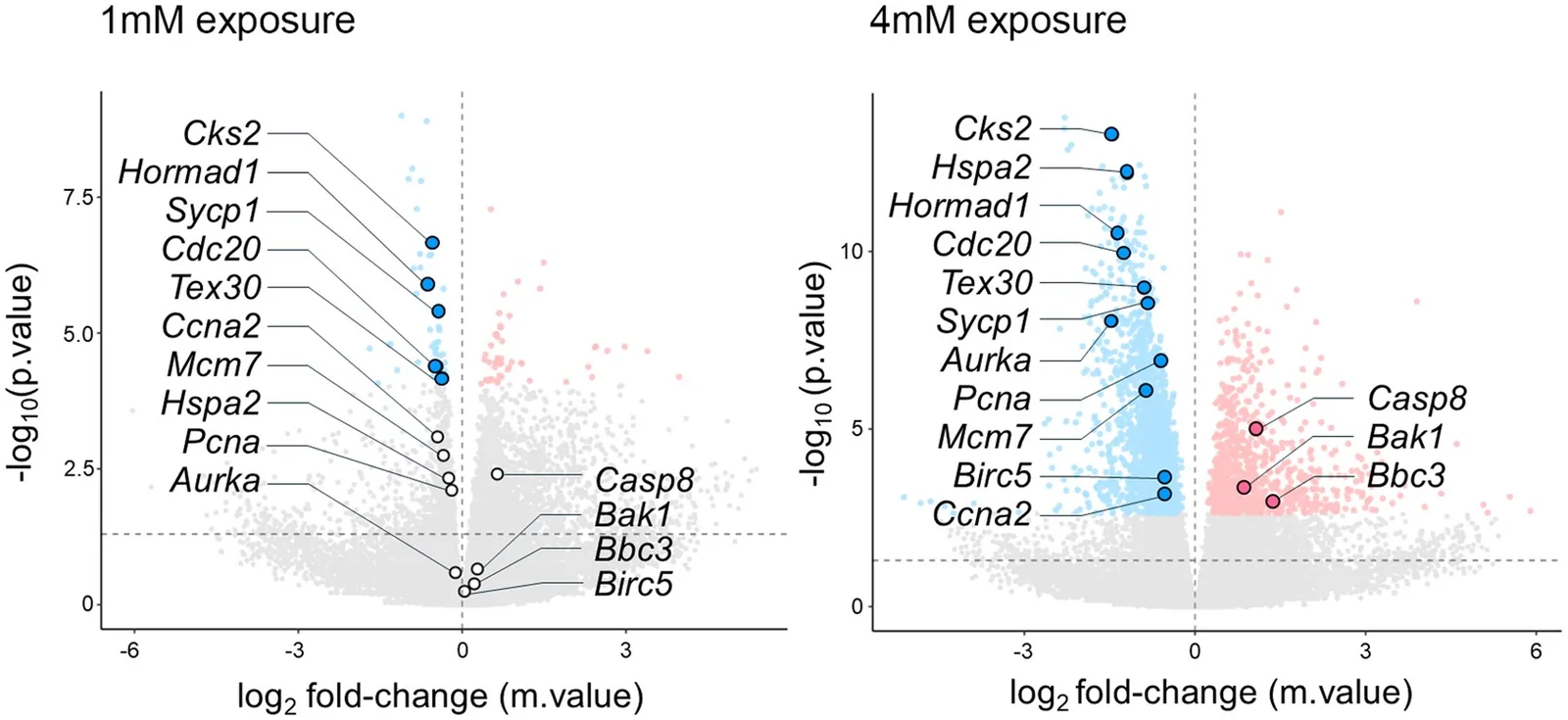
Transcriptomic responses captured in the testis organ culture system after exposure. RNA-seq analysis of biologically independent cultured testis tissues (vehicle control, 1 mM MAA, and 4 mM MAA; *n* = 4 to 5 per group). Volcano plots showing differential gene expression at 24 h under each exposure condition relative to vehicle controls (FDR = 0.05). Upregulated genes are shown in red, downregulated genes in blue, and nonsignificant genes in gray. Among representative genes selected based on their relevance to AOP-defined key events, genes with significant changes are highlighted, whereas selected genes without significant changes are indicated by white circles. GO enrichment analysis summarizing pathway-level responses is shown in Table 1.

Transcriptomic responses following exposure are summarized in Fig. 6. To characterize early molecular perturbations captured within the testis organ culture system under defined exposure conditions, transcriptomic analysis was performed following exposure to MAA as a mechanistically well-characterized reference compound. Gene expression profiles were compared among vehicle-treated controls and tissues exposed to 1 mM or 4 mM MAA.

MAA exposure induced a concentration-dependent increase in the number of differentially expressed genes (DEGs), with more pronounced transcriptional responses at 4 mM (Fig. 6). Using an FDR-adjusted *P*-value threshold of 0.05, a total of 96 DEGs were identified at 1 mM MAA and 2,626 DEGs at 4 mM. In the volcano plots, a subset of genes involved in cell cycle regulation and meiotic processes (e.g. *Cks2, Cdc20, Sycp1, Hormad1, Ccna2*, and *Tex30*) showed statistically significant changes already at 1 mM, despite the absence of detectable alterations in GFP expression, histological architecture, or apoptotic markers, indicating that molecular perturbations precede overt cellular and tissue-level effects.

At 4 mM, transcriptional alterations became more extensive, including additional changes in cell cycle- and meiosis-related genes (e.g. *Mcm7* and *Hspa2*) and induction of apoptosis-related genes (e.g. *Casp8, Bbc3/PUMA*, and *Bak1*), consistent with downstream cellular and tissue-level responses at this exposure level. At this higher concentration, the observed downregulation of meiotic genes may partly reflect changes in cellular composition resulting from pachytene spermatocyte depletion, in addition to transcriptional perturbation at the single-cell level.

Gene Ontology (GO) enrichment analysis further supported these patterns (Table 1). At 4 mM, GO terms related to cell cycle regulation and apoptotic signaling were significantly enriched, whereas GO terms associated with meiotic progression were selectively enriched among downregulated genes (Table S1). At 1 mM, GO terms associated with cell cycle regulation and meiotic progression were already significantly enriched, suggesting early molecular perturbations prior to overt cellular loss. At 4 mM, suppression of mitotic and meiotic processes became more pronounced. For GO enrichment analysis, DEGs were separated into upregulated and downregulated gene sets, and only genes with available GO annotations were included. Therefore, the denominators shown in Table 1 represent the number of GO-annotated DEGs within each regulated gene set rather than the total DEG count.

**Table 1.** GO enrichment analysis of differentially expressed genes identified in the testis organ culture system after exposure.

AOP key event	Representative GO term (FDR < 0.05)	Representative genes (with references)	GO enrichment (GO DEGs/GO-annotated DEGs, *q*)
KE1: disruption of cell-cycle regulation	Mitotic cell cycle phase transition (↓)	*Cks2* (Frontini et al. 2012) *Cdc20* (Yu 2007) *Aurka* (Hirota et al. 2003) *Ccna2* (Silva Cascales et al. 2021) *Pcna* (Moldovan et al. 2007) *Mcm7* (Nallamshetty et al. 2005)	1 mM (down): 3/37, *q* = 1.83E−06 4 mM (down): 71/1,582, *q* = 2.18E−14
Suppression of meiotic progression	Meiotic cell cycle (↓)	*Hspa2* (Eddy 1999) *Hormad1* (Shin et al. 2010) *Sycp1* (Horisawa-Takada et al. 2021) *Tex30* (Sun et al. 2025b)	1 mM (down): 8/37, *q* = 4.08E−05 4 mM (down): 88/1,582, *q* = 1.12E−31
KE3: activation of apoptosis	Regulation of apoptotic signaling pathway (↑)	*Casp8* (Kominami et al. 2012) *Bbc3/PUMA* (Han et al. 2001) *Bak1* (Youle and Strasser 2008) *Birc5/Survivin* (Ambrosini et al. 1997)	1 mM (up): Not significant 4 mM (up): 36/786, *q* = 9.80E−07

GO terms related to cell cycle regulation and apoptotic signaling, corresponding to key events described in AOP 212, as well as GO terms related to meiotic progression, which were observed under the present experimental conditions, were significantly enriched among DEGs identified under 1 mM and/or 4 mM exposure conditions. Representative genes associated with each GO term are shown. Complete DEG lists and full GO enrichment results are provided in Table S1. ↓, downregulated; ↑, upregulated. The total numbers of DEGs (FDR < 0.05) were 96 at 1 mM and 2,626 at 4 mM. Gene ratios were calculated using GO-annotated DEGs within each regulated gene set rather than the total DEG count.

KE1 and KE3 correspond to key events described in AOP 212. Suppression of meiotic progression is included as an additional process identified under the present experimental conditions.

Together, these transcriptomic patterns support suppression of meiotic progression as a candidate intermediate event linking early molecular perturbations to downstream spermatocyte depletion within this organ culture system.

Overall, transcriptomic profiling identified coordinated changes involving cell cycle regulation, suppression of meiotic progression as a candidate intermediate event, and activation of apoptotic pathways within a single testis organ culture framework.

These findings do not imply direct reproduction of in vivo AOP sequences, but rather reflect the sequential molecular changes observed under the experimental conditions employed in this study, using MAA as a mechanistically well-characterized reference compound.

### Residual MAA retention in conventional PC cultures

**Fig. 7.**
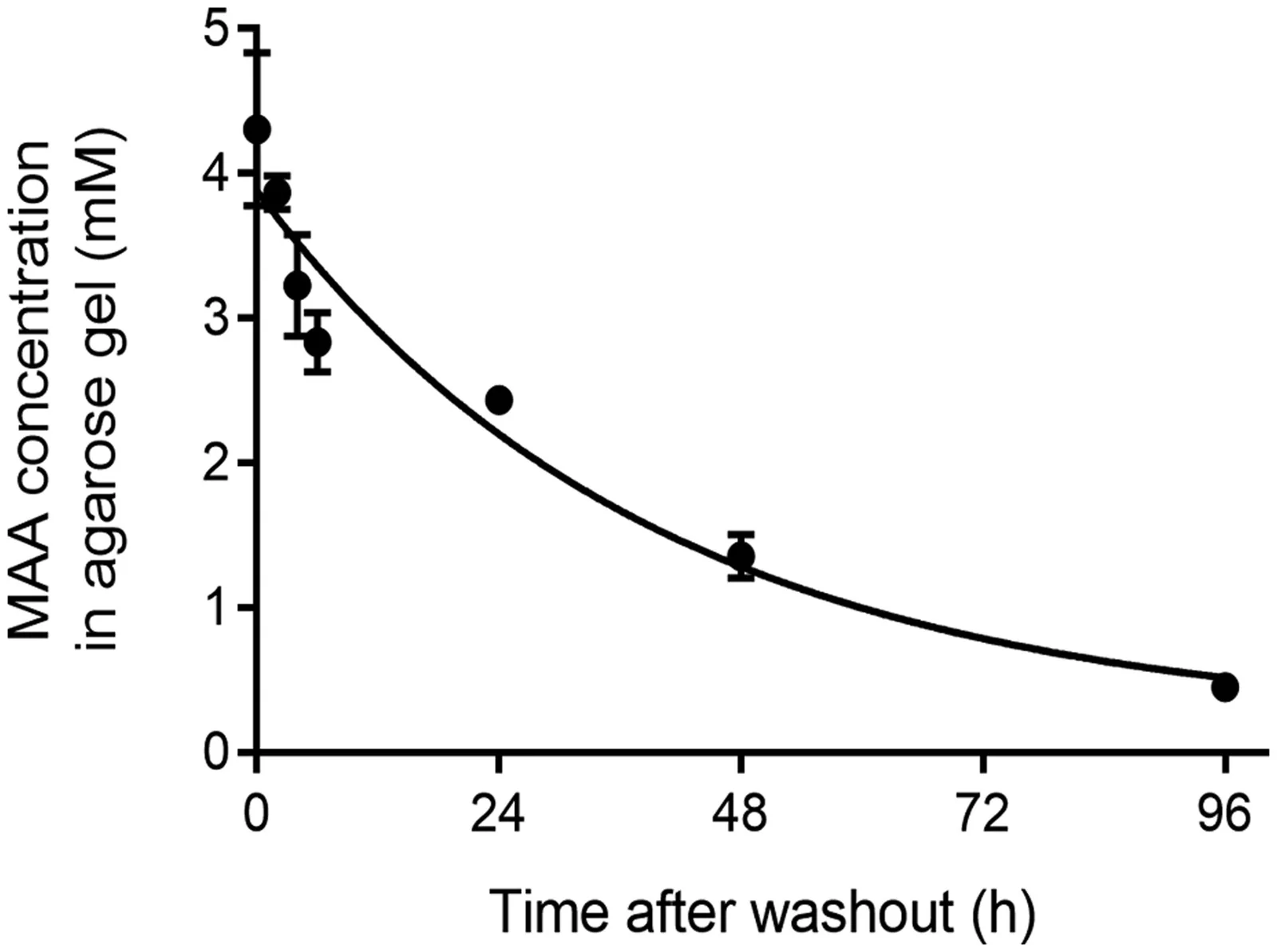
Time course of residual MAA concentration in agarose gels. Agarose gel blocks containing 4 mM MAA were washed according to the procedure described in the Materials and Methods, following the same washing schedule used in the recovery assay (0, 2, 4, and 6 h and 1, 2, and 4 d postexposure, with three washes at each time point). Residual MAA was extracted from the gels and quantified by LC-MS/MS. Data are shown as mean ± SD (*n* = 3). Despite repeated washing, MAA persisted in the agarose gels and gradually declined over time.

Before conducting recovery experiments, we quantified residual MAA levels in agarose gel blocks following nominal exposure termination. LC-MS/MS analysis revealed that measurable concentrations of MAA persisted in agarose-supported PC cultures despite medium replacement and repeated washing procedures (Fig. 7). These findings indicate that effective exposure may continue after nominal termination under conventional PC conditions. Given that accurate assessment of recovery requires precise control of exposure termination, the persistence of MAA within the agarose matrix may influence the interpretation of recovery dynamics under conventional PC conditions. Therefore, the membrane-supported PC (msPC) configuration was designed to minimize compound retention and to enable rapid transition to compound-free conditions.

### Assessment of recovery processes in testis organ culture using the msPC method

To assess recovery processes under precisely controlled postexposure conditions, we evaluated a modified PDMS-ceiling method incorporating a membrane support (msPC method), which allows rapid removal of test compounds at the end of exposure.

In conventional PC cultures, residual MAA persisted within the agarose gel after medium replacement and washing (Fig. 7), suggesting that effective overexposure may continue after treatment cessation. The msPC method was therefore designed to minimize compound retention and enable prompt transition to compound-free conditions (Figs 1b and 8a).

**Fig. 8.**
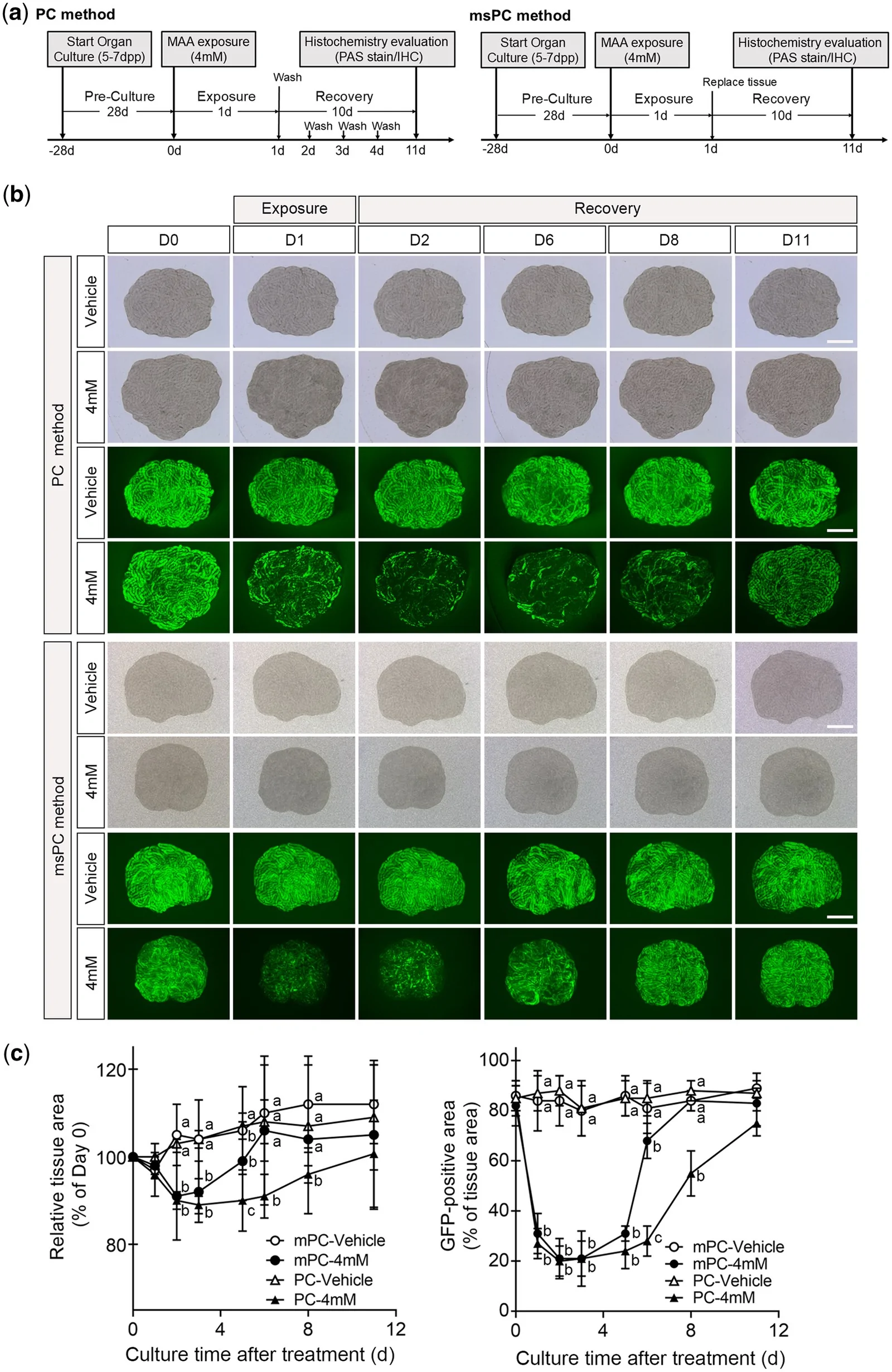
Assessment of recovery dynamics in testis organ culture using PC and msPC configurations. (a) Experimental workflow for exposure (1 to 4 mM MAA, 24 h) and subsequent recovery under controlled postexposure conditions; tissues were collected on day 11 for histological evaluation. (b) Representative bright-field and GFP fluorescence images obtained before exposure (0 h), after 24 h exposure to 4 mM MAA, and during recovery. Recovery occurred earlier in msPC cultures than in conventional PC cultures. (c) Quantitative analysis of projected tissue area and GFP-positive area during recovery, normalized to pre-exposure values (0 h = 100%). Recovery proceeded significantly faster in msPC cultures. Data are mean ± SD (*n* = 8); different letters indicate *P* < 0.05. (d) PAS-stained sections after recovery showing restored tissue morphology in both PC and msPC cultures (*n* = 3). (e) Immunohistochemical analysis showing spermatocyte numbers at recovery day 10. No significant differences were detected compared with vehicle controls. Data are shown as box-and-whisker plots (*n* = 5). SYCP3, green; γH2AX, magenta; DAPI, gray. Scale bars: 40 µm (histology/IHC), 20 µm (higher magnification).

**Fig. 8.**
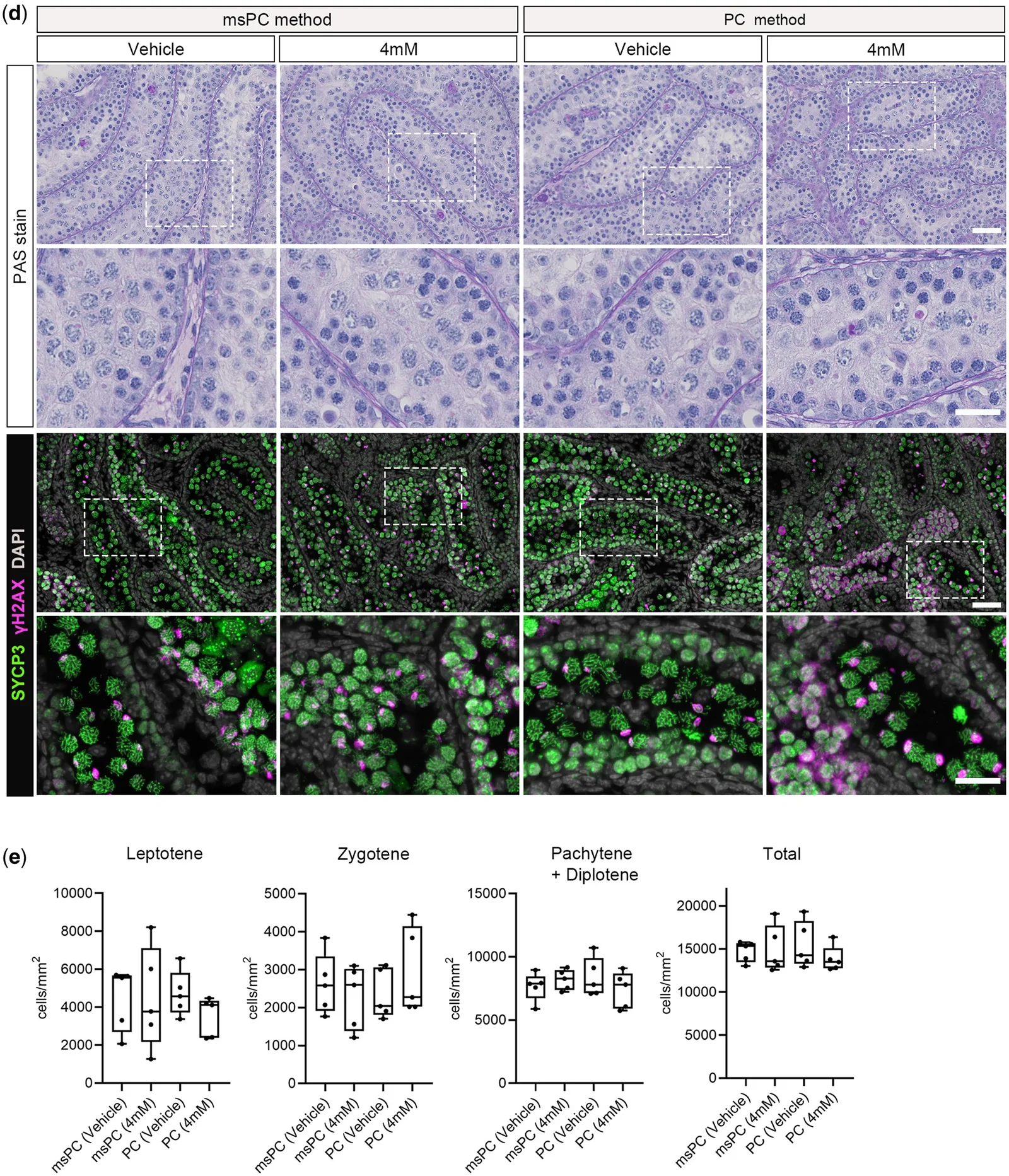
Continued.

For recovery experiments, a 4-mM exposure condition was selected to induce a robust toxic response prior to exposure termination. Quantitative analysis of bright-field and GFP fluorescence images showed that, in both PC and msPC cultures, GFP-positive area and tissue area transiently decreased after exposure and subsequently recovered (Fig. 8b and c). Although no change in tissue area was detected at 24 h after exposure (Fig. 2), longitudinal observation revealed a slight but statistically significant transient tissue atrophy at later time points. Notably, recovery of both GFP-positive area and tissue area occurred earlier in msPC cultures than in conventional PC cultures.

After the recovery period, histological and immunohistochemical analyses showed that tissue morphology and spermatocyte numbers were comparable to those of vehicle-treated controls (Fig. 8c-e). Together, these results suggest that the msPC method reduces unintended overexposure after treatment cessation and enables assessment of recovery dynamics in testis organ culture systems under well-controlled ex vivo conditions.

## Discussion

In this study, we established an AOP-aligned mouse testis organ culture workflow based on the PC method and examined whether sequential molecular, cellular, and tissue-level changes relevant to testicular toxicity can be captured within a single ex vivo system under defined exposure conditions. Using MAA as a mechanistically well-characterized reference compound, we integrated live GFP readouts, histology, phase-resolved immunohistochemistry, apoptosis assessment, and transcriptomics to position early key events relative to subsequent tissue-level outcomes and to evaluate recovery under controlled postexposure conditions. The novelty of this study lies in a platform-level approach that enables multi-level mechanistic information to be aligned within the same experimental framework, addressing a key limitation of previous ex vivo testis culture studies that primarily focused on single endpoints. This study does not introduce a new culture technique, but rather demonstrates how the existing PC system can be configured as an AOP-aligned framework integrating multi-level endpoints with controlled recovery assessment. The flattened configuration of the PC method reduces diffusion distances and helps maintain relatively uniform oxygen and nutrient supply, thereby minimizing culture-associated background variability and facilitating clearer interpretation of toxicant-induced changes. These design features enabled temporal organization of AOP-defined key events within a controlled experimental configuration.

**Fig. 9.**
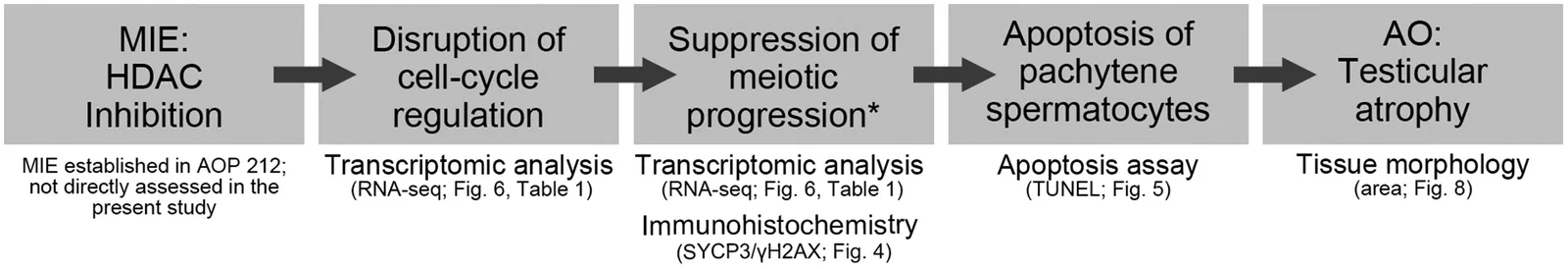
Alignment of endpoints assessed in the present ex vivo organ culture study with the established adverse outcome pathway (AOP 212) for MAA-induced testicular toxicity. The molecular initiating event (histone deacetylase [HDAC] inhibition) is established in AOP 212 and was not directly evaluated in this study. Downstream key events, including disruption of cell-cycle regulation, suppression of meiotic progression,* and apoptosis of pachytene spermatocytes, were evaluated using the indicated endpoints. Testicular atrophy represents the adverse outcome (AO) defined in AOP 212 and was assessed histologically in the present study. *Suppression of meiotic progression is shown as a putative intermediate process observed under the present experimental conditions.

To provide a structured overview of the established AOP framework for MAA-induced testicular toxicity and to clarify how the endpoints assessed in the present study align with this framework, a schematic representation is shown in Fig. 9.

From a mechanistic perspective, the sequence of molecular and cellular changes observed in this system is consistent with the AOP describing histone deacetylase inhibition leading to testicular atrophy (Tanabe et al. 2021). This AOP proposes a cascade involving disruption of cell cycle regulation, suppression of meiotic progression, induction of apoptosis, and subsequent tissue atrophy.

The temporal sequence of these key events was established through in vivo investigations. In the present study, these molecular and cellular events were experimentally aligned and examined within a controlled organ culture system under defined exposure conditions. Importantly, rather than attempting to reproduce this pathway in vivo, the present study demonstrates that downstream molecular and cellular key events defined in AOP 212 can be positioned and examined within a single organ culture system under identical experimental conditions.

In the present organ culture system, selective depletion of pachytene spermatocytes and increased apoptotic germ-cell responses were observed under identical exposure conditions prior to detectable tissue atrophy. These findings are consistent with the sequence of key events described in AOP 212. Importantly, these phase-resolved and apoptosis-related changes were characterized within a single experimental framework, allowing their temporal relationships to be examined under defined ex vivo conditions. However, these results should be interpreted as being aligned with, rather than establishing, the mechanistic sequence described in vivo.

In the present framework, endpoints across biological levels were acquired under controlled and consistent exposure conditions, exposure duration, and sampling window. At 24 h, GFP-defined spermatogenic impairment and phase-resolved spermatocyte loss were detectable without measurable changes in tissue area, enabling early spermatogenic disruption to be positioned temporally ahead of overt atrophy within the same organ culture system. Importantly, this temporal relationship was defined under identical experimental conditions within the same organ culture system, thereby allowing early and later events to be examined in relation to each other within an AOP-informed framework. Transcriptomic profiling further indicated that molecular perturbations can be detected even when overt cellular- and tissue-level changes are not yet apparent. Notably, at 1 mM exposure, transcriptional suppression of cell cycle- and meiosis-related pathways was observed in the absence of detectable GFP reduction, histological alteration, or apoptotic response, supporting the interpretation that molecular key events precede downstream cellular manifestations.

Interpretation of Gene Ontology enrichment in this study focused on pathway-level directional tendencies rather than on absolute statistical metrics. Enrichment of cell cycle- and meiotic process-related terms among downregulated genes at lower exposure levels suggests early molecular disruption, whereas more pronounced suppression of these pathways and activation of apoptosis-related processes at higher exposure levels is consistent with subsequent spermatocyte loss and tissue atrophy. This pattern supports suppression of meiotic progression as a candidate intermediate event linking early molecular perturbations to downstream cellular outcomes within this system. Within this integrated setting, spermatocyte depletion was not treated as a stand-alone histological observation but was further specified by meiotic phase. Phase-resolved immunohistochemistry demonstrated preferential loss of pachytene spermatocytes, whereas spermatogonia and Sertoli cell populations were comparatively preserved. In parallel, TUNEL analysis indicated that apoptosis accompanies spermatocyte loss at exposure levels where cellular depletion is evident. Aligning these measurements within a single experimental framework enables an experimentally traceable linkage among early readouts that are often examined separately across different models or studies.

Understanding the reversibility of toxicity is essential for interpreting the dynamic nature of testicular injury and for regulatory decision-making, as reversibility represents a critical modifier of toxicity and adversity alongside the magnitude and duration of observed changes (International Council for Harmonisation of Technical Requirements for Pharmaceuticals for Human Use (ICH) 2011; Hukkanen et al. 2023). In this study, we therefore focused on enabling a more precise evaluation of recovery dynamics by addressing limitations inherent to conventional organ culture configurations. We demonstrated that agarose-supported PC cultures can retain test compounds after nominal exposure termination, resulting in prolonged effective exposure and delayed recovery. Such retention may confound interpretation of recovery dynamics by effectively extending exposure beyond the intended termination point. In contrast, the membrane-supported PC (msPC) configuration minimized unintended compound retention, allowing stricter temporal control of exposure and a more accurate assessment of recovery dynamics. Together, these findings highlight that control of postexposure conditions is a critical design element for interpreting reversibility in ex vivo testis organ culture systems.

On the other hand, the PC-based organ culture system preserves the basic seminiferous tubule architecture; however, under culture conditions, it does not necessarily achieve the same level of tissue maturation or structural integrity as observed in vivo. In particular, although this culture system supports the progression of meiosis, spermatogenic efficiency is markedly lower than that in vivo; therefore, the application of strict seminiferous tubule staging based on conventional in vivo histopathological evaluation is limited. In the present study, meiotic phase classification of spermatocytes was performed using γH2AX and SYCP3 immunostaining criteria confirmed in vivo, enabling phase-specific analysis within the experimental framework. Elongated spermatids were observed in testes collected at 5 to 7 d postpartum and cultured for 4 wk. Given that mouse spermatogenesis requires ∼30 d, the developmental timeline in the present study corresponds to ∼5 wk of age, at which elongated spermatids could be expected to emerge.

Several limitations should be acknowledged. Bulk RNA-seq reflects averaged gene expression across heterogeneous cell populations and cannot fully distinguish cell-intrinsic transcriptional changes from shifts in cellular composition. At higher doses, the observed downregulation of meiotic genes may not solely reflect alterations in meiotic progression at the single-cell level. Rather, it may partially represent the depletion of pachytene spermatocytes and subsequent reduction of round spermatids, as demonstrated by histological evidence of apoptosis. Therefore, interpretation of bulk RNA-seq data in this context should consider the potential impact of altered cellular composition. Integration of single-cell transcriptomics, spatial transcriptomics, and computational deconvolution approaches would further refine cell type-resolved interpretation of early molecular events (Hansen et al. 2025; Lu et al. 2025; Sun et al. 2025a). In addition, while the organ culture system supports key aspects of spermatogenic differentiation, it does not recapitulate systemic pharmacokinetics or metabolic activation, which may limit applicability to certain classes of compounds (Bell et al. 2018; Davies et al. 2020).

Evaluation of the mechanistic validity of this system requires determining whether the observed changes reflect responses associated with specific cellular stages. In the present study, selective depletion of pachytene spermatocytes—an established target cell stage of MAA—was observed, whereas no significant reduction was detected in spermatogonia or Sertoli cell numbers (Fig. S2). In addition, TUNEL-positive signals were predominantly localized within the seminiferous epithelium rather than the interstitial compartment (Fig. 5). These findings suggest that the observed effects reflect stage-dependent cellular responses rather than widespread nonspecific cytotoxicity.

Postmeiotic germ cell populations were not quantitatively evaluated because differentiation efficiency at these stages remains limited in the organ culture system. However, PAS-stained sections (Fig. 3) indicated the presence of round and elongated spermatids under the 24-h exposure conditions, with no apparent acute depletion. This observation is consistent with the known stage-specific mechanism of MAA toxicity, which primarily targets pachytene spermatocytes during early exposure. Future incorporation of refined analytical strategies may enable more systematic evaluation of postmeiotic cell populations.

Multiple tissue samples can be obtained from a single intact animal, enabling evaluation of multiple exposure conditions and hierarchical endpoints under controlled experimental conditions, while contributing to reduction of animal use. This efficiency contributes to the *Reduction* principle of the 3Rs by minimizing the number of intact animals required for mechanistic evaluation (Stokes 2015; Brannen et al. 2016). Moreover, continued refinement of spermatogenesis reconstruction using tissues derived from nonreproductive livestock may, in the longer term, support partial *Replacement* of animal testing (Kanbar et al. 2022; Ibtisham et al. 2023). In addition, the present approach is well-suited for early-stage safety assessment of candidate compounds that require mechanistic clarity and precise control of exposure conditions, particularly when the available quantity of test compounds is limited.

Despite these limitations, the present study provides a proof-of-concept for an AOP-aligned evaluation framework using a single reference compound (MAA). The organ culture system supports analysis of multiple germ cell populations and may therefore be adaptable to toxicants targeting different germ cell types or mechanisms of action. However, systematic validation using compounds with distinct modes of action remains an important future direction. The present findings provide a foundation for integrated evaluation of molecular, cellular, and tissue-level changes in testicular toxicity within a single experimental system and are expected to contribute to the further development of NAM (New Approach Methodologies)-based approaches for testicular toxicity assessment.

## Supplementary Material

kfag037_Supplementary_Data

## Data Availability

All data supporting the findings of this study are available in the paper and Supplementary data. RNA-seq data were deposited in the Gene Expression Omnibus (GEO) under the accession number GSE314869.
