# Supplementary figures and images for: An adverse outcome pathway-aligned ex vivo mouse testicular organ culture platform for mechanistic integration of multi-level endpoints with recovery assessment
Source: Toxicol Sci. 2026 Mar 28;209(4):kfag037. doi: 10.1093/toxsci/kfag037 (PMC13092378; doi:10.1093/toxsci/kfag037)

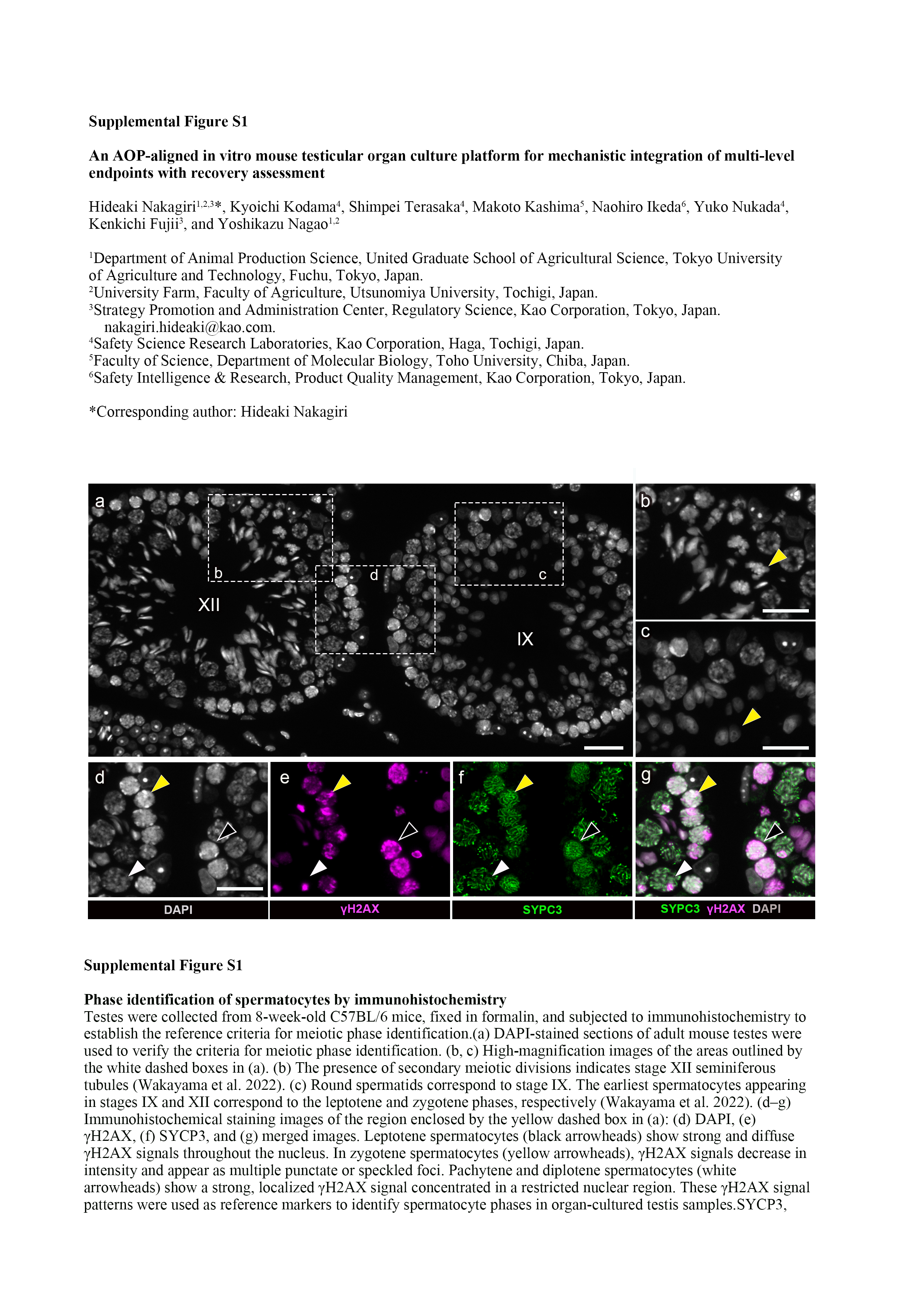

Supplement: kfag037_Supplementary_Data [file kfag037_supplementary_data.zip › 03-Apr-2026_074337_sFig1.tif]

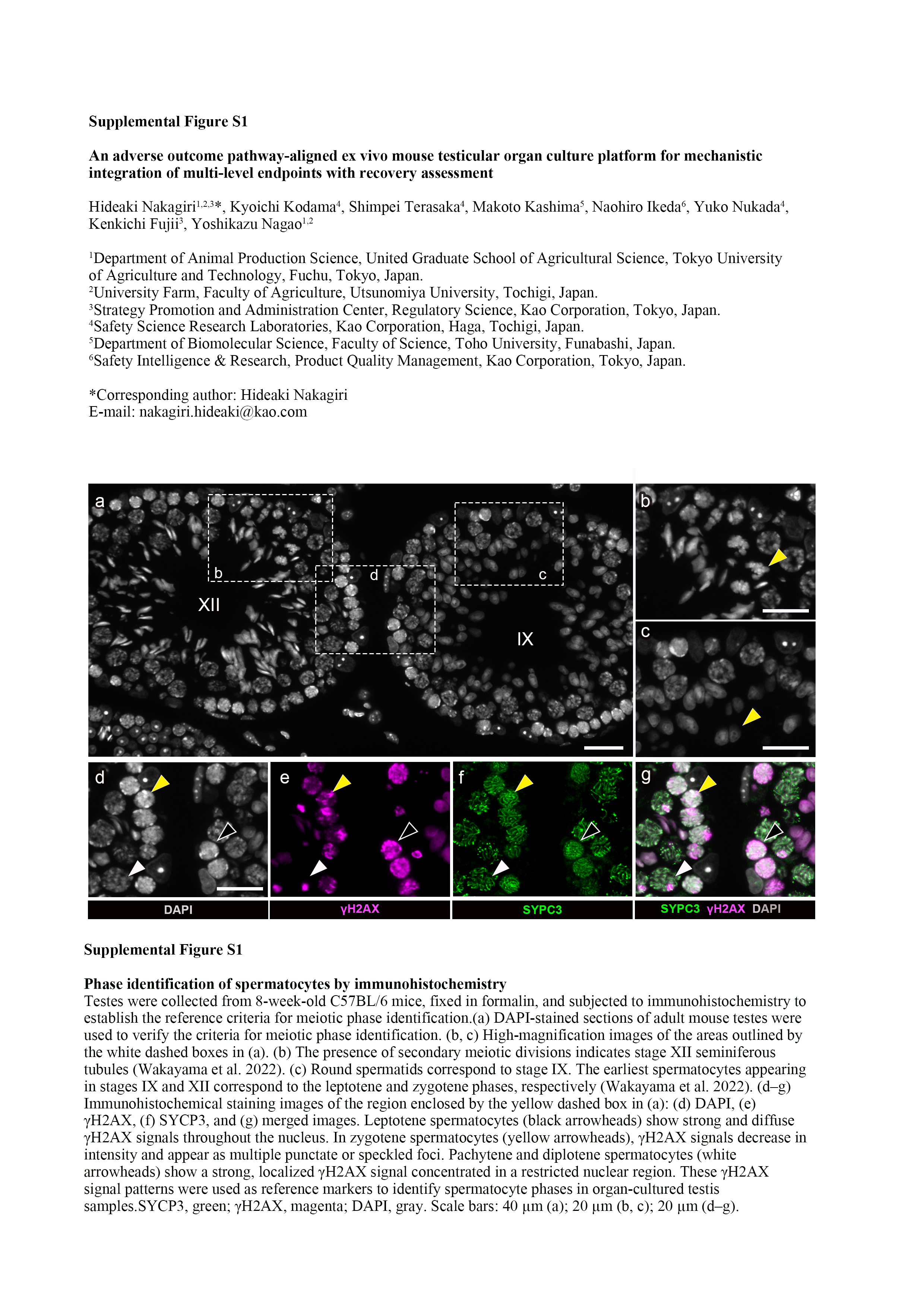

Supplement: kfag037_Supplementary_Data [file kfag037_supplementary_data.zip › sFig1.tif]

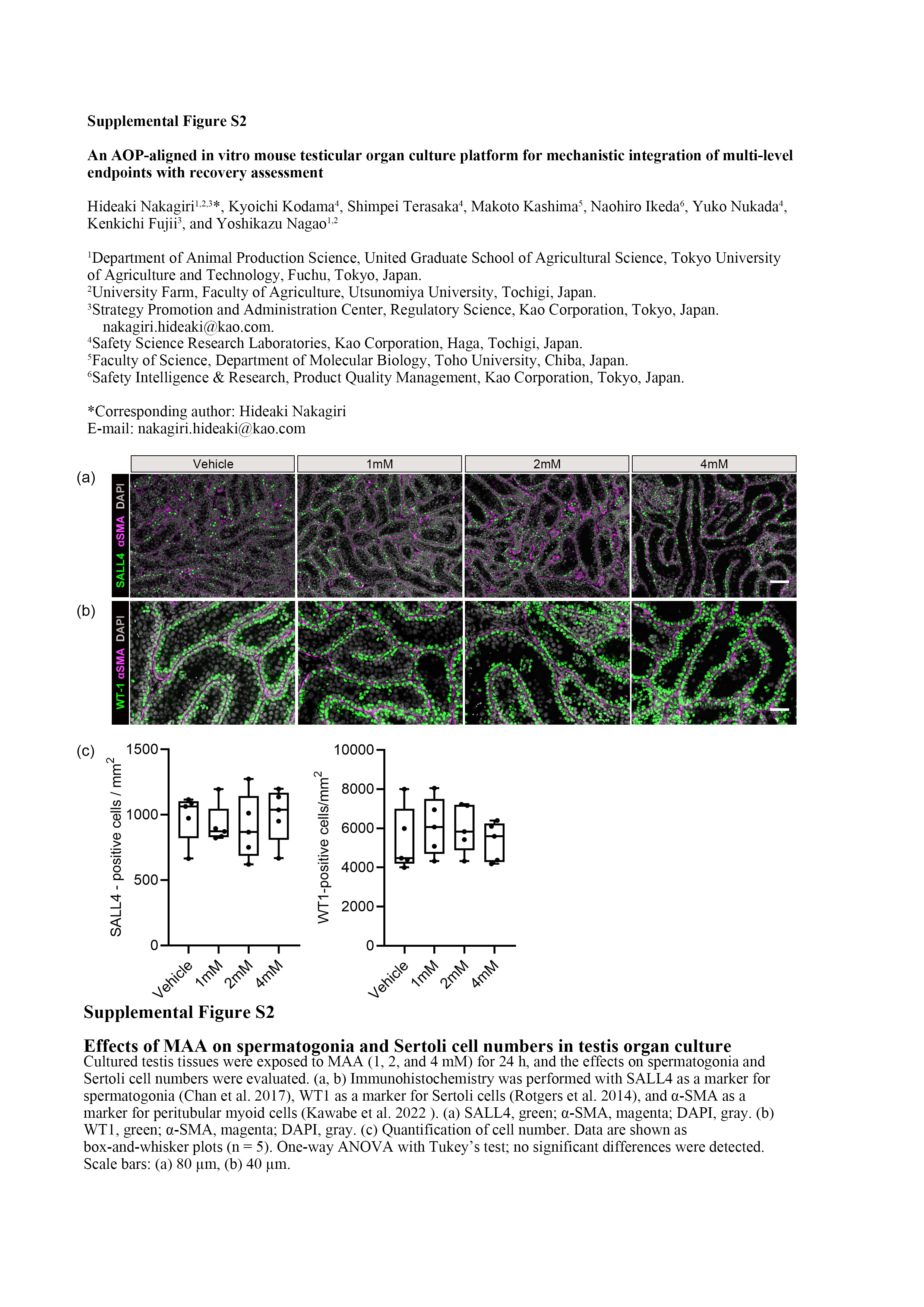

Supplement: kfag037_Supplementary_Data [file kfag037_supplementary_data.zip › 03-Apr-2026_074336_sFig2.tif]

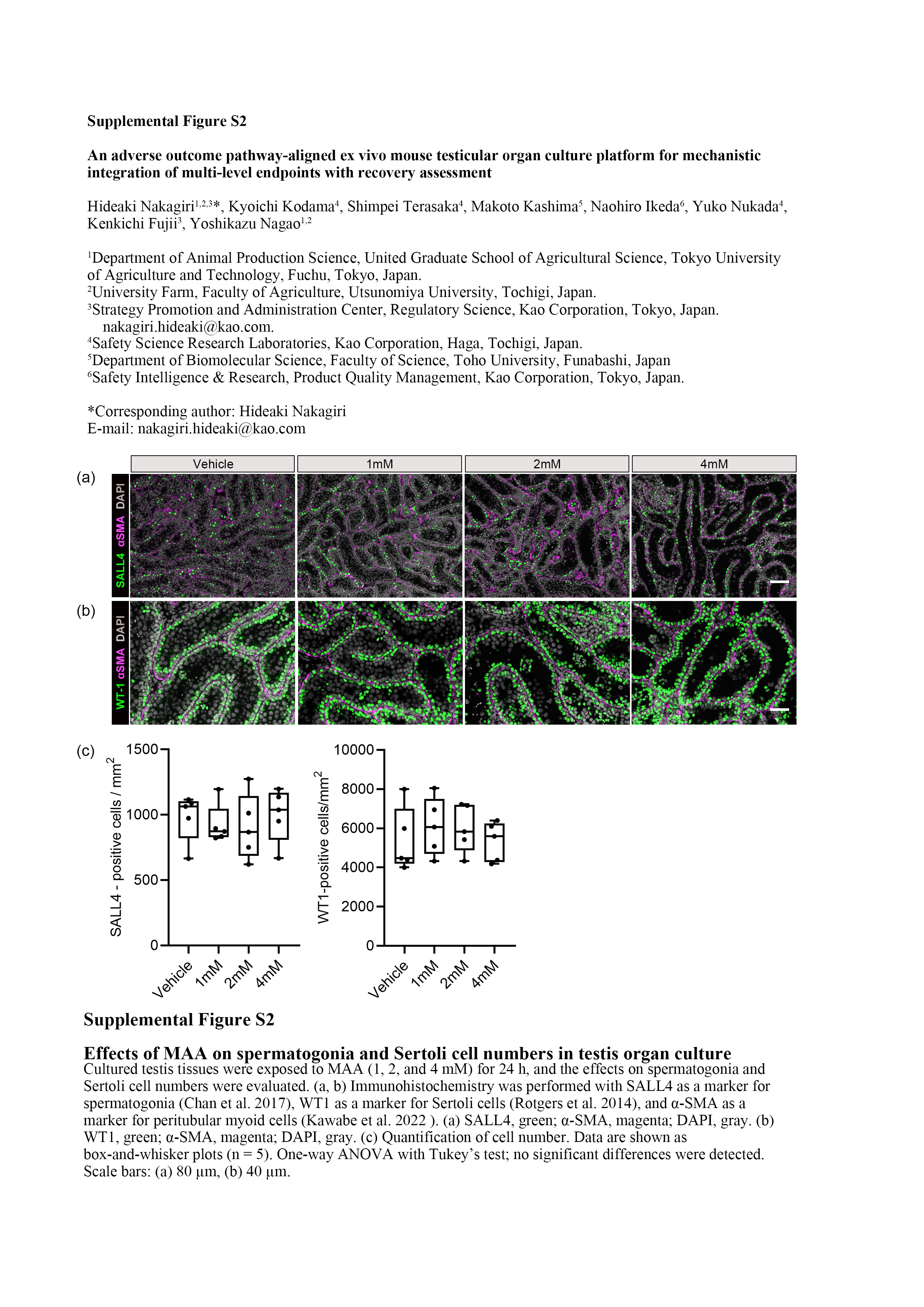

Supplement: kfag037_Supplementary_Data [file kfag037_supplementary_data.zip › 07-Apr-2026_014951_sFig2.tif]
